# Pulsed Laser Deposition Derived Bioactive Glass-Ceramic Coatings for Enhancing the Biocompatibility of Scaffolding Materials

**DOI:** 10.3390/ma13112615

**Published:** 2020-06-08

**Authors:** Ruxandra-Ioana Schitea, Alexandru Nitu, Andreea-Aurelia Ciobota, Andrei-Lucian Munteanu, Irina-Madalina David, Dana Miu, Mina Raileanu, Mihaela Bacalum, Cristina Busuioc

**Affiliations:** 1 Faculty of Medical Engineering, University POLITEHNICA of Bucharest, RO-011061 Bucharest, Romania; ruxandra.schitea@stud.fim.upb.ro (R.-I.S.); alexandru.nitu0805@stud.fim.upb.ro (A.N.); aurelia.ciobota@stud.fim.upb.ro (A.-A.C.); andrei.munteanu2501@stud.fim.upb.ro (A.-L.M.); irina.david@stud.fim.upb.ro (I.-M.D.); 2 National Institute for Laser, Plasma and Radiation Physics, RO-077125 Magurele, Romania; dana_miu2001@yahoo.com; 3 Horia Hulubei National Institute of Physics and Nuclear Engineering, RO-077125 Magurele, Romania; rmina@nipne.ro (M.R.); bmihaela@nipne.ro (M.B.); 4 Faculty of Applied Chemistry and Materials Science, University POLITEHNICA of Bucharest, RO-011061 Bucharest, Romania

**Keywords:** glass-ceramic, coatings, pulsed laser deposition, bioactivity, biocompatibility

## Abstract

The purpose of this work was to propose and evaluate a new composition for a bioactive glass-ceramic starting from the well-known 45S5 commercial product. Thus, we developed a modified version, including MgO, an oxide that turned out to induce superior mechanical properties and improved biological response. This had the following molar percentages: 46.1% SiO_2_, 2.6% P_2_O_5_, 16.9% CaO, 10.0% MgO, and 24.4% Na_2_O. The precursor alkoxides and nitrates were processed by a standard sol-gel technique, resulting in a glass-ceramic target, suitable for laser ablation experiments. Combeite (Na_2_Ca_2_Si_3_O_9_) was identified as a main crystalline phase within the calcined sol-gel powder, as well as in the case of the target sintered at 900 °C. The thin films were deposited on silicon substrates, at room temperature or 300 °C, being subsequently characterized from the material point of view, as well as in terms of bioactivity in simulated conditions and biocompatibility in relation to human fibroblast BJ cells. The investigations revealed the deposition of nanostructured glassy layers with a low proportion of crystalline domains; it was shown that a higher substrate temperature promoted the formation of surfaces with less irregularities, as a consequence of material arrangement into a shell with better morphological homogeneity. The complex elemental composition of the target was successfully transferred to the coatings, which ensured pronounced mineralization and a stimulating environment for the cell cultures. Thereby, both samples were covered with a thick layer of apatite after immersion in simulated body fluid for 28 days, and the one processed at room temperature was qualified to be the best in relation to the cells.

## 1. Introduction

Tissue engineering represents an interdisciplinary field that uses and correlates aspects from biology, chemistry, materials science, engineering, medicine, and so on, in order to achieve materials for tissue and organ regeneration or restoration [1]. Due to the worldwide increasing incidence of different trauma injuries and debilitating health affections, such as cancer or diabetes, there is a major need for tissue engineering products [2].

As the scientific research and development field, tissue engineering has been firstly recognized since the early 1993s, and its market growth rises more and more yearly [1]. According to the “Tissue Engineering 2018–2028: Technologies, Markets, Forecasts” market report, the market of tissue engineering products is about to exceed $4.8 billion by the year 2028 [3].

Bioglass^®^ or 45S5 was first developed by Larry Hench in 1969, and it represents a commercially available product, composed of 45 wt% SiO_2_, 24.5 wt% CaO, 24.5 wt% Na_2_O, and 6.0 wt% P_2_O_5_ [4]. It is based on the ability of SiO_2_ as a glass network former and exhibits several key compositional features that are responsible for the associated bioactivity: low SiO_2_ content, high Na_2_O and CaO content, and high CaO/P_2_O_5_ ratio. Besides its difficulty of processing and inherent brittleness, this material displays biocompatibility and bone-bonding capability due to the formation of a carbonate-substituted hydroxyapatite-like layer on the glass surface in contact with the body fluid, as a result of chemical degradation and ions release [5]. 45S5 glass has been extensively studied in recent decades and is being processed with different dimensionalities and for various purposes. In the form of particles, it has been incorporated in glass ionomer cements, which has enhanced their mechanical and remineralizing properties [6], or it has been dispersed in natural rubber fibers, leading to hybrid membranes with good thermal and mechanical properties [7]. Deposited as coatings, 45S5 bioglass has increased the corrosion resistance of Ti6Al4V alloy [8], while, as scaffolds coated with dual Mg–Zn-loaded hydroxyapatite nanoparticles, it has shown enhanced biological performance [9]. In the doped state, it may be successfully used as bone defect filler, even with more effectiveness than the pristine counterpart [10].

Bioglass 45S5 has generated a new class of inorganic biocompatible materials. Following the tests performed by different researchers, it has resulted in a number of advantages that make these systems suitable for the medical field [11]. One of the most important properties is that of biocompatibility; this is given by the similarity with hydroxyapatite at the compositional level [12]. Hydroxyapatite (Ca_5_(PO_4_)_3_(OH)) is the hydroxyl endmember of the apatite group, with the carbonated calcium-deficient variant individualizing as the main component of bones and teeth [13]; its chemical resemblance to the hard tissue minerals guarantees the occurrence of a direct bond to the natural bone, without an intervening fibrous layer, namely, important implications on implants osseointegration, especially when containing valuable cations [14].

Moreover, these glassy materials favor the healing process by in vivo release of calcium, phosphorus, and silicon ions. From a mechanical point of view, they display high compressive strengths, but the limitations are triggered by the intrinsic fragility [15]. The antibacterial effect is another important property, which is ensured when the release of alkaline ions occurs [16].

The incorporation of various elements through ion doping is a widely approached method for improving the mechanical characteristics, biochemical behavior, and therapeutic properties of bioactive glasses [17]. Magnesium (Mg) is one of the most important trace elements associated with the biological apatites, having an important role in the maintenance and development of bone matrix [18], as well as being the co-factor of various enzymes and taking part in more than 300 chemical reactions [17]. Moreover, it is involved in the promotion of catalytic reactions, mineral metabolism [19], biocompatibility, and osteoconductivity over time [18]. Strontium (Sr) has the ability to promote osteoblast function and osteogenesis [20] while inhibiting bone resorption and osteoclast activity [19]. Oral supplements of strontium ranelate have been used for treating osteoporosis, with beneficial effects on in vivo bone formation [20]; thus, bioglasses are ideal candidates for Sr delivery in bone tissue engineering, owning to their homogenous composition [21]. Some studies have shown that cerium (Ce) substitution induces an antioxidant activity in such systems (that generally have negligible antioxidant qualities) [22] while promoting osteointegration, bone formation, and reduction of reactive oxygen species levels [23].

The sol-gel method is the best-known wet-chemical method for preparing materials and nanomaterials of oxide or composite nature, supposing several steps—hydrolysis, condensation, drying, and calcination [24]. This preparation route is commonly used due to numerous advantages, like simplicity and low cost, addressability to different types of compositions, a wide range of precursors, and successful implementation for extremely varied applications—from optics to mechanics and from electronics to medicine [25,26,27].

Pulsed laser deposition (PLD) is a physical vapor deposition technique where a high-power pulsed laser beam is focused inside a vacuum chamber to strike a target of the material that is to be deposited on a selected substrate [28]. The versatility of PLD is that there is almost no restriction on the source material to be used [29]. The thickness of the deposition is in the range of 10–500 nm [28], while the main advantage derives from the laser-assisted material removal mechanism [30]: a photon interaction to create an ejected plume of material from any target, conceptually simple, fast for nanometric thicknesses, clean due to the employed energy, and cost-effective.

In this paper, we reported on the synthesis and characterization of bioactive glass-ceramic thin films belonging to SiO_2_‒P_2_O_5_‒CaO‒MgO‒Na_2_O oxide system, with the aim of proposing a new type of magnesium incorporating coatings for enhancing the performance of implantable medical elements.

## 2. Materials and Methods

### 2.1. Target Fabrication

In order to prepare a mineral powder having the following composition: 46.1% SiO_2_, 2.6% P_2_O_5_, 16.9% CaO, 10.0% MgO, and 24.4% Na_2_O (molar percentages), tetraethyl orthosilicate (TEOS, Si(OC_2_H_5_)_4_, 98%, Aldrich, St. Louis, MO, USA), triethyl phosphate (TEP, PO(OC_2_H_5_)_3_, ≥99%, Merck, St. Louis, MO, USA), calcium nitrate tetrahydrate (Ca(NO_3_)_2_·4H_2_O, 99–102%, Merck), magnesium nitrate hexahydrate (Mg(NO_3_)_2_·6H_2_O, 99%–102%, Merck), and sodium nitrite (NaNO_2_, ≥99%, Riedel-de Haën, Charlotte, NC, USA) were employed as reagents, all of them with high purity. A standard sol-gel procedure was applied; the details of similar approaches could be found in previous works [31,32]. After the gelation, aging, and drying procedures, the resulting powder was calcined at 650 °C for 2 h. Then, it was granulated and shaped like a disk through uniaxial pressing in a stainless steel mold with a 25 mm inner diameter, in this way, obtaining the target green body. This was eventually sintered at 900 °C for 10 h, in order to provide a compromise between fairly good densification and a mandatory hindering of crystallization.

### 2.2. Thin Films Deposition

The deposition experiments were performed with an Nd-YAG laser (EKSPLA model NL301HT, EKSPLA, Vilnius, Lithuania), having a pulse duration of about 5 ns and using a pulse repetition rate of 10 Hz. Nanosecond lasers are used for pulsed laser deposition (PLD) in most cases due to the energy per pulse, which is several orders of magnitude higher than that for pico-and femtosecond lasers, leading to a higher film deposition rate. A UV wavelength of 355 nm was selected due to the well-known advantages of UV laser ablation. Although the laser is also equipped with a module for operation at 266 nm, the low energy per pulse at this wavelength does not permit the ablation of targets with a large ablation threshold, such as ceramic ones. The laser beam was focused onto the target using a 320 nm focal length lens and was incident on the target at an angle of about 45° from the target normal. The focused beam had dimensions of about 1 × 0.5 cm^2^ on the target, leading to an energy density of about 1.5 J/cm^2^ for the 73–74 mJ pulse energies employed. The oriented silicon substrates (plates of 1 × 1 cm^2^, which were subsequently cut into 4 identical samples) were placed 40 mm from the target, parallel to its surface. The depositions were made in a vacuum chamber under a controlled oxygen atmosphere with 100 mTorr pressure. Based on our experience in the field and the findings made during the optimization trials, two thin films were grown, both using 33,000 pulses: Film 1-deposited at room temperature, and Film 2-deposited at 300 °C substrate temperature. The number of ablation pulses, determined in previous experiments, was chosen in order to ensure that the films were thick enough to remain stable during the biological evaluation.

### 2.3. Physicochemical Characterization

The complex thermal analysis of the precursor dried gel was recorded using a Shimadzu DTG-60 equipment (Shimadzu Corporation, Kyoto, Japan), in air, up to the temperature of 650 °C, with a heating rate of 10 °C/min. The morphology achieved in all stages was studied by scanning electron microscopy (SEM) with an FEI Quanta Inspect F microscope (FEI Company, Hillsboro, OR, USA) equipped with an energy-dispersive X-ray spectroscopy (EDX) probe. A Thermo Scientific Nicolet iS50 spectrophotometer (Thermo Fisher Scientific, Waltham, MA, USA) was used for studying the chemical bonding and grouping within materials through Fourier-transform infrared spectroscopy (FTIR), the wavenumber ranging between 400 and 4000 cm^−1^, with 4 cm^−1^ resolution. A Shimadzu XRD 6000 diffractometer (Shimadzu Corporation, Kyoto, Japan) was employed for analyzing the phase composition of the intermediate powder and final target through X-ray diffraction (XRD), while a PANalytical Empyrean diffractometer (Malvern Panalytical, Royston, UK) was operated at grazing incidence in the case of the coated samples, both with Ni-filtered Cu, Kα radiation (*λ* = 1.54 Å), 2*θ* ranging between 10 and 80°, with a scan speed of 2°/min.

### 2.4. Biological Evaluation

The mineralization capacity in simulated body fluid (SBF) was estimated by immersion for 28 days, at 37 °C, in a testing solution prepared according to Kokubo [33], followed by compositional and morphological characterization of the newly formed layer with the help of the earlier mentioned techniques and equipment.

Cell metabolic activity was assessed by employing the MTT assay, as described previously [34]. The human fibroblast BJ cells (ATCC, CRL-2522) were placed on the surfaces found in 24 well plates at a density of 20,000 cells/well. As a control, the cells were grown on glass slides in similar conditions. They were first grown for 48 h, after which the medium and the discs were removed, and MTT was added to each well (at a final concentration of 1 mg/mL). The cells were further incubated for 4 h when the medium was removed, and dimethyl sulfoxide (DMSO) was added to dissolve the formed crystals. The solution absorbance was recorded at *λ* = 570 nm using the plate reader Mithras LB 940 (Berthold, Bad Wildbad, Germany). Cell metabolic activity was determined with the formula: Cell viability = (Corrected absorbance of treated cells/Corrected absorbance of control cells) × 100.

After incubation for 48 h with the samples, the morphological changes induced in the cells found around them were observed under a CKX53 bright-field microscope (Olympus, Tokyo, Japan), employing a 10× objective and photographed with a WAT-902H camera (Watec, Saint-Lambert-la-Potherie, France).

The morphological modifications were also investigated by fluorescence microscopy. The actin filaments were stained using Phalloidin-FITC (Sigma-Aldrich, Saint Louis, MO, USA), while the nucleus with Hoechst 33,342 (Invitrogen, Karlsruhe, Germany), as briefly described. The cells were grown on the surfaces placed in 24 well plates, in similar conditions, as described above. After 48 h, they were washed with phosphate-buffered saline (PBS), fixed with 4% formaldehyde, washed again with PBS, and permeabilized with 0.1% Triton X-100 in PBS. The cells were washed again with PBS, and the two fluorescent dyes were added together on the cells and left in the dark, at room temperature, for 1.5 h. Finally, they were washed with PBS and fixed with FluorSave^TM^ (Merck, Darmstadt, Germany). The fluorescence images were taken with a confocal microscope (Andor DSD2 Confocal Unit, Singapore) mounted on an Olympus BX-51 epifluorescence microscope, employing a 40× objective. The images were recorded using an appropriate DAPI/Hoechst filter cube (excitation filter 390/40 nm, dichroic mirror 405 nm, and emission filter 452/45 nm) in the case of the nucleus and a suitable GFP/FITC filter cube (excitation filter 466/40 nm, dichroic mirror 488 nm, and emission filter 525/54 nm) in the case of actin filaments. The two channels were further pseudo-colored and overplayed, employing ImageJ software (version 1.53a, Madison, WI, USA).

## 3. Results and Discussion

### 3.1. Powder and Target Characterization

**Figure 1:**
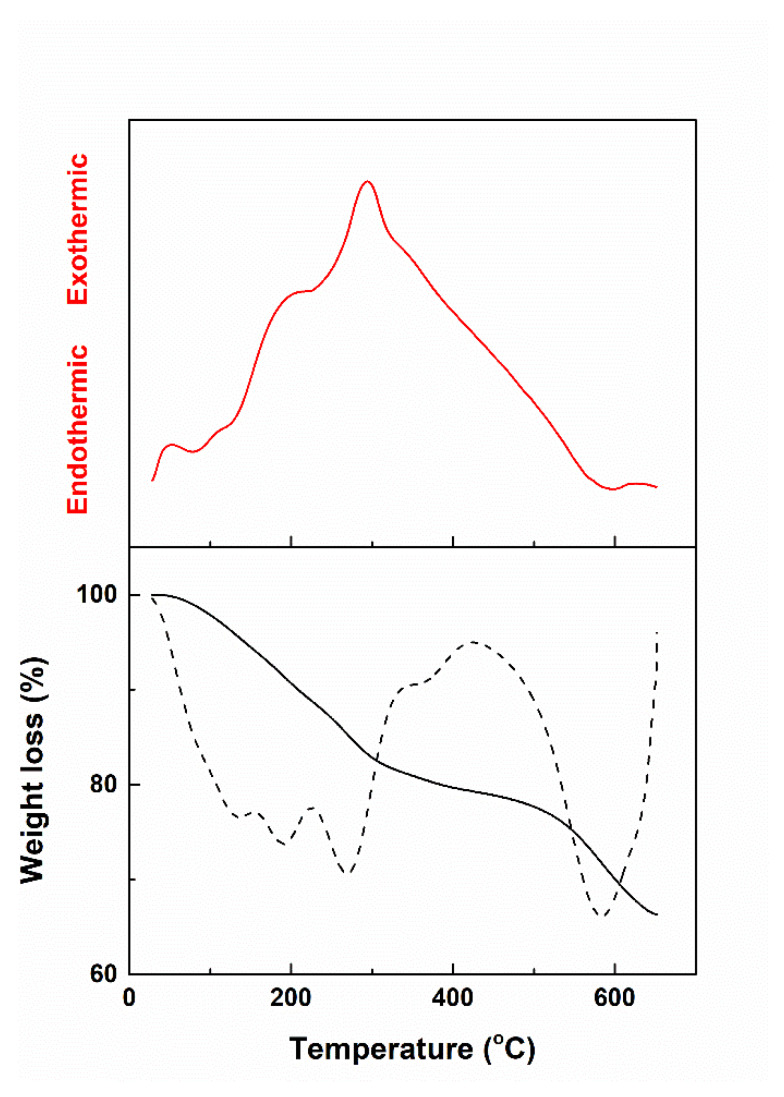
Complex thermal analysis of the dried gel (solid black curve—thermogravimetric analysis, dashed black curve—derivative of the thermogravimetric analysis with respect to time, red curve—differential thermal analysis).

A thermal analysis was performed up to 650 °C on the dried gel in order to determine the appropriate calcination temperature, the temperature at which most of the gas generating processes would be completed, while the nucleation and growth of any crystalline phase would be hindered. As it can be seen in Figure 1, the sample suffered several weight loss steps, some of them overlapped, but the most important two centered at 270 and 585 °C; these were related to the burning of the organic groups from the alkoxides and decomposition of the nitrate groups from the corresponding reagents, facts also confirmed by the sequence of thermal effects. Overall, a third of the sample was lost when the temperature reached the maximum value, while the aspect of the curves indicated a certain tendency of thermal stabilization above 650 °C. In these conditions, considering our experience in the field as well, it was decided to calcine the dried gel at 650 °C for 2 h, even if there was a possibility of remaining organic traces in a proportion of a few percentages.

**Figure 2:**
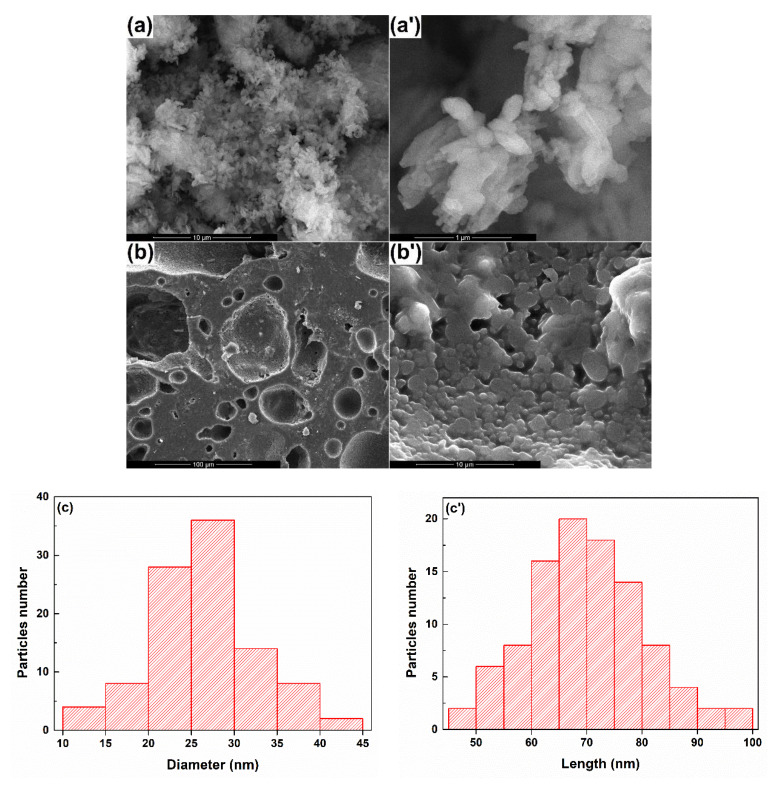
SEM images of: (**a**-10,000× and **a’**-100,000×) the powder and (**b**-1000× and **b’**-10,000×) the target and (**c** and **c**’) statistical analyses of particles diameter and length.

The calcined powder was converted into a target by applying pressing and sintering procedures so that both the shape and the texture fulfilled the requirements imposed by the PLD equipment in terms of diameter, height, and densification. Thus, the green body was pressed at 150 MPa and sintered at 900 °C for 10 h, with the dimensions of the final disc as follows: 25.0 mm diameter, 4.5 mm height. The microstructure can be visualized in Figure 2, together with the morphology of the primary powder. The last one was composed of particles with different shapes, ranging from elongated to quasi-spherical (Figure 2a,a’); their size also variated, with the values for the mean diameter and length of individual particles determined by a statistical analysis performed on 100 particles: 26 nm average particle diameter and 96 nm average particle length (Figure 2c,c’). As far as the target was concerned, the micronic grains were relatively well packed in a three-dimensional body; however, some large quasi-spherical pores were visible at lower magnification and small branched channels in between the rounded grains at higher magnification (Figure 2b,b’). Indeed, the degree of densification could be improved, but the increase of the sintering temperature or time was not an option in our case since this would trigger an accentuated crystallization.

**Figure 3:**
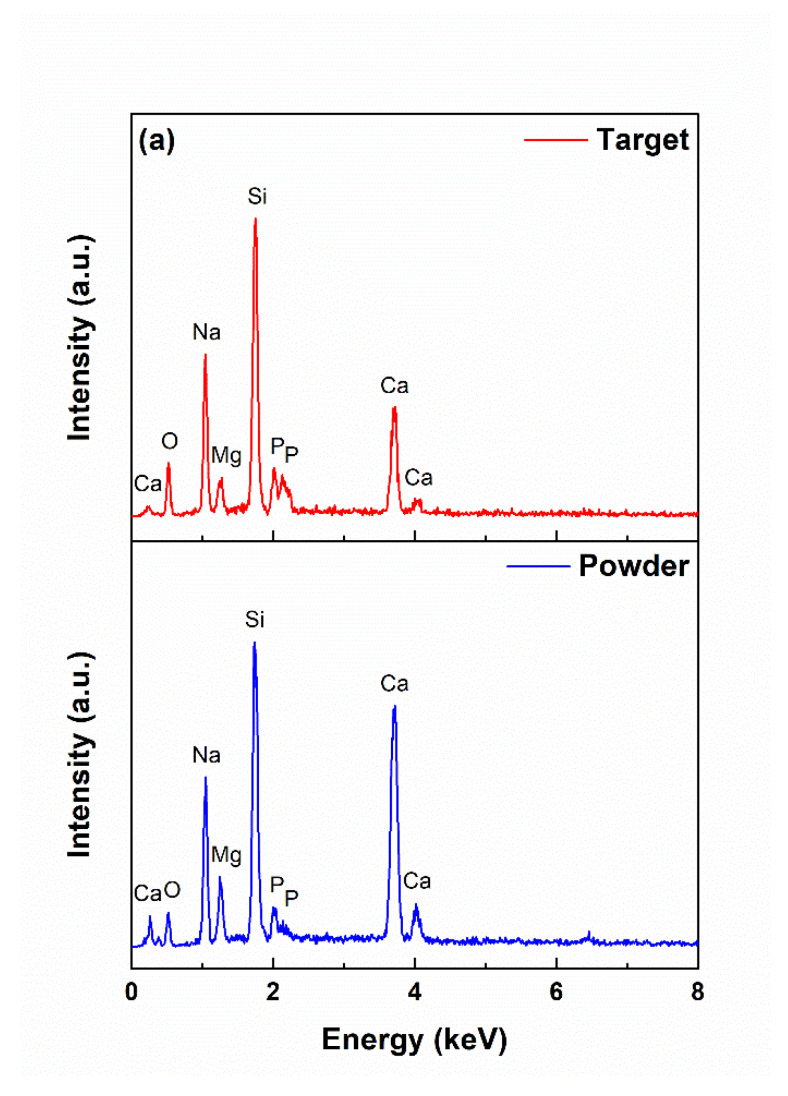

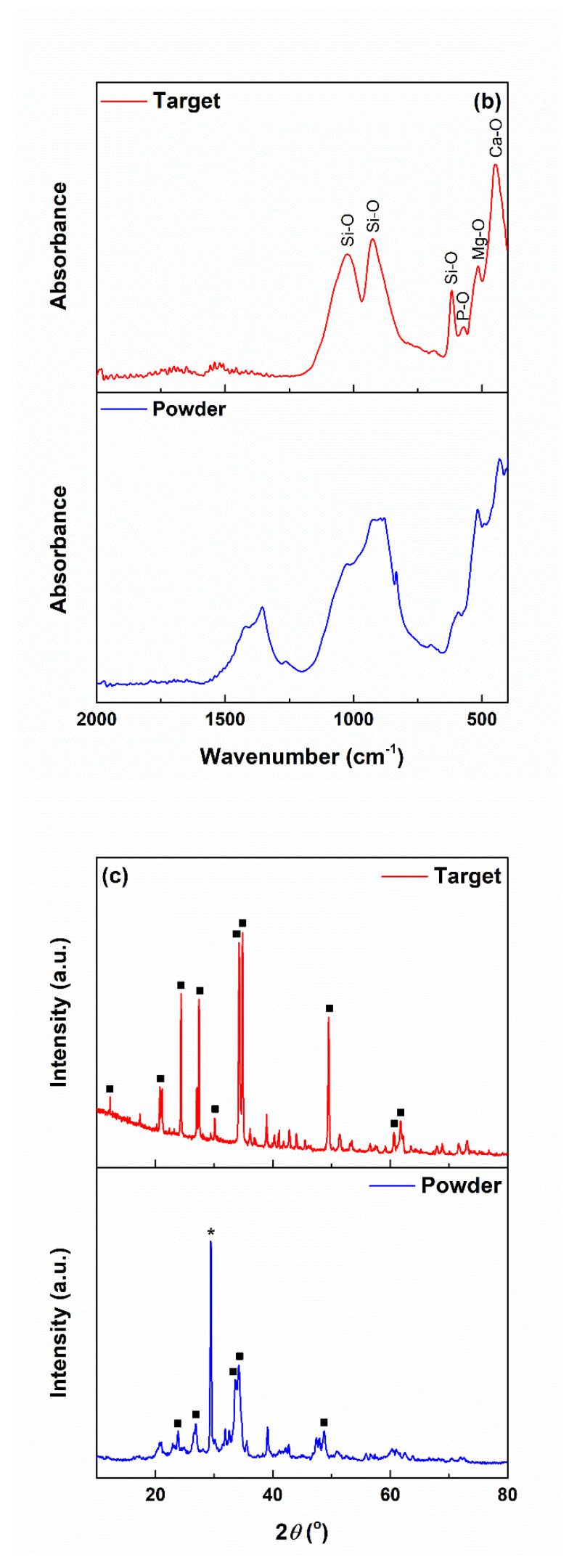
(**a**) EDX spectra, (**b**) FTIR spectra, and (**c**) XRD patterns of the powder and target. Square indicates Na_4_Ca_4_Si_6_O_18_, and star shows SiO_2_.

Considering the elemental composition, all targeted elements could be found in the two synthesis stages—powder and target: large amounts of silicon (Si), sodium (Na), and calcium (Ca), as well as small amounts of magnesium (Mg) and phosphorus (P), the rations from the theoretical recipe being quite well respected (Figure 3a). This validated that the designed oxide composition was maintained in the target. Figure 3b shows how the previously mentioned elements joined in order to generate chemical bonds or groups. If, in the case of the powder, the situation is not plain yet, the FTIR spectra of the target exhibit well-outlined bands attributed to Si–O, Ca–O, Mg–O, and P–O vibrational phenomena [31,35,36]. Going further, to the identification of the crystalline compounds, the XRD patterns (Figure 3c) indicated a mixture of silicon dioxide (SiO_2_) with monoclinic structure (ICDD 00-083-1833) and sodium-calcium silicate (Na_2_Ca_2_Si_3_O_9_ or Na_4_Ca_4_Si_6_O_18_, known as combeite) with hexagonal structure (ICDD 00-079-1089) in the case of the powder, which evolved towards the same sodium calcium silicate as a major phase in the case of the target. Moreover, it was observed that all diffraction maxima were significantly shifted to the right compared to the information from the database, which could suggest a pronounced degree of doping with other elements from the system itself. The crystallinity level was high even from the powder stage, meaning that for the selected composition, it was not possible to inhibit the mechanisms of nucleation and growth.

Chen et al. [37] fabricated scaffolds starting from 45S5 bioglass powder; they found that Na_2_Ca_2_Si_3_O_9_ crystalline phase provided good mechanical support temporarily, while promoting bioactivity, and, at later stages, it biodegraded with a tailorable rate as a function of crystallinity. In this context, other researchers have tried to control the formation of combeite in order to tailor the crystallinity of 45S5 glass-ceramics over a wide range [38]. Another study has concluded that Na_2_Ca_2_Si_3_O_9_ induces hydroxyapatite formation after soaking in simulated body fluid for 1 day, which reveals good bioactivity and better hydroxyapatite forming ability than partially crystallized 45S5 bioglass [39]. On the other hand, Cabal et al. [40] concluded that combeite was also responsible for an obvious antibacterial capability, meaning that a combeite-containing glass-ceramic was effective in inhibiting film formation without the need of loading with antibiotics or metallic nanoparticles. Moreover, when compositing a bioactive glass-ceramic with Na_2_Ca_2_Si_3_O_9_ as a major crystalline phase with gelatine in the form of the scaffold, the results have revealed an enhanced osteogenic ability and good cell adhesion properties [35]. All these suggest that combeite is a valuable compound in tissue engineering applications.

### 3.2. Coatings’ Physicochemical Characterization

The described target was employed as a source of material in two laser ablation experiments, which led to the deposition of thin films on the top of silicon substrates. Unlike IR or visible wavelengths, UV wavelengths led to limited thermal effects at the target surface, as well as stronger target absorption, allowing stoichiometric removal of target complex material and good film uniformity and morphology (limiting the formation of droplets on the film surface, for example).

**Figure 4:**
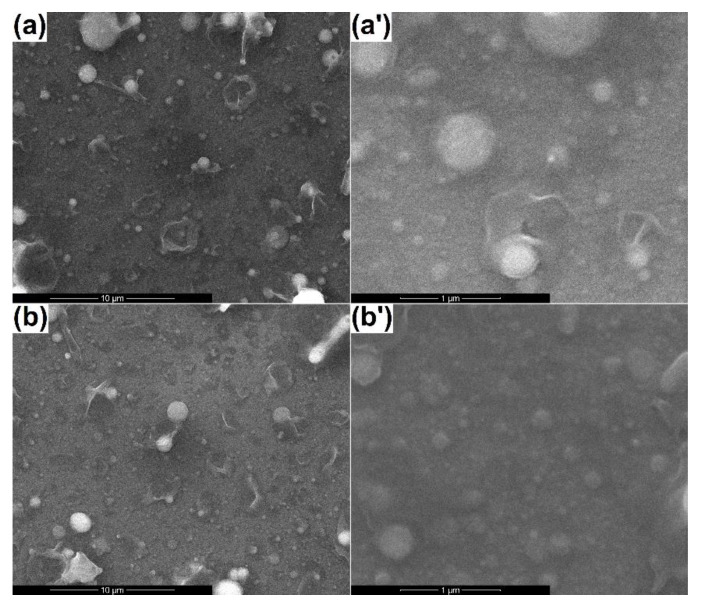
SEM images of the coatings processed at (**a**-10,000× and **a’**-80,000×) room temperature and (**b**-10,000× and **b’**-80,000×) 300 °C.

The aspect of the newly created surfaces is displayed in Figure 4. Specific droplets, predominantly spherical in shape and of different sizes, could be seen for both samples. Apart from these, more complex structures, sometimes individualized as open-cavity distorted spheres or ribbon-like twisted fragments, resulted after bombarding the surface with aggregates containing plasma, were present on the entire investigated areas; these represented consequences of the target porous microstructure. Besides this rough appearance, the layers were continuous and nanostructured, with grains below 50 nm, and they seemed relatively homogenous in thickness. The influence of the substrate temperature could be quantified, taking into account the surface quality; thus, it seemed that an energy supplement coming from the substrate helped to a certain extent the material arrangement when hitting the support, reducing the number of polymorphic formations or decreasing their dimension.

**Figure 5:**
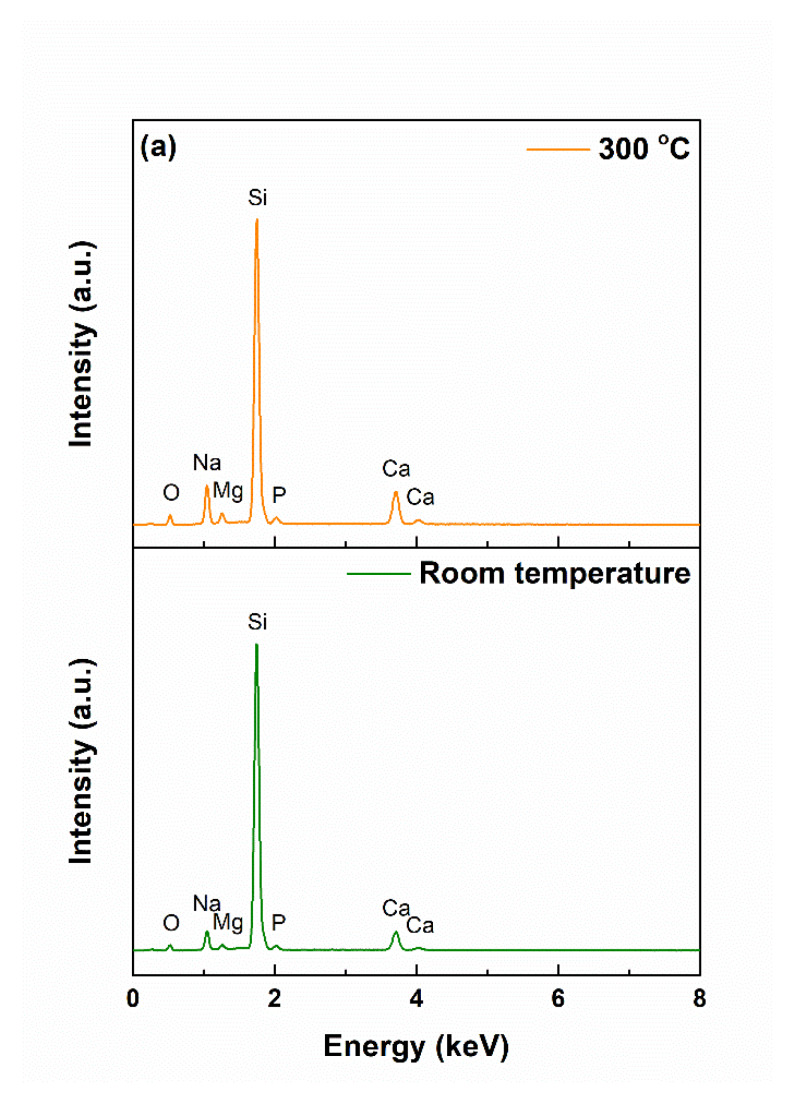

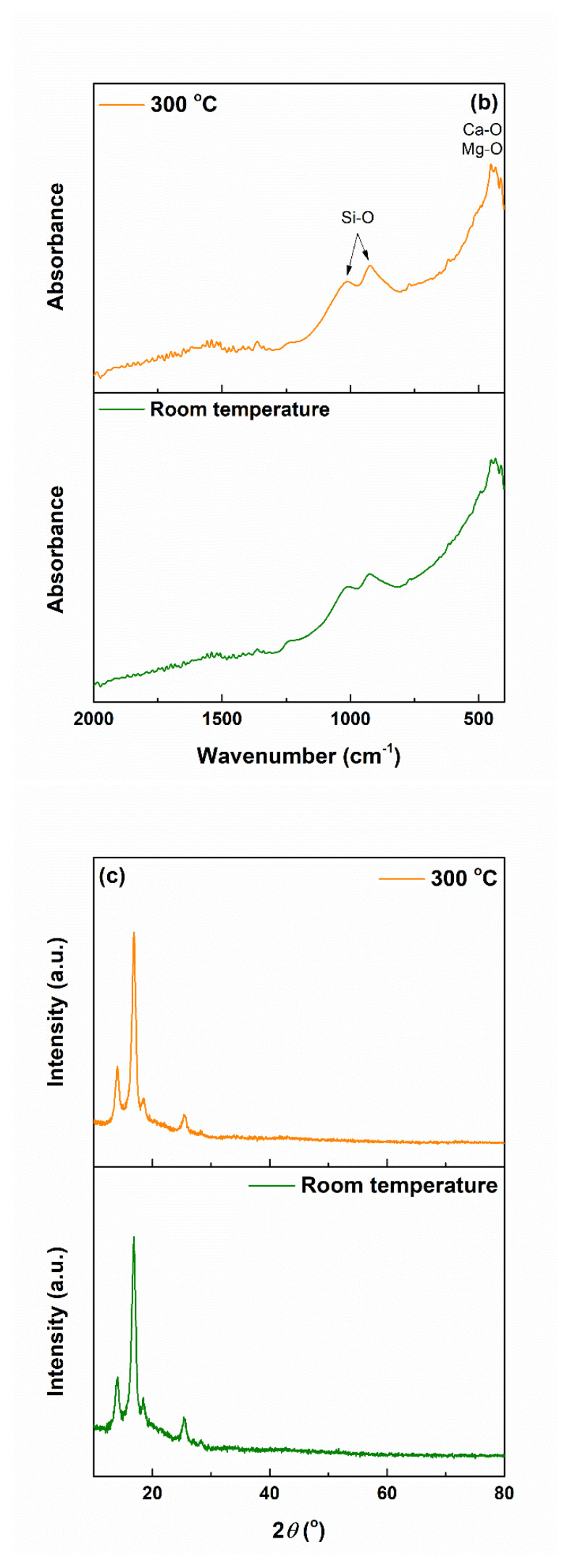
(**a**) EDX spectra, (**b**) FTIR spectra, and (**c**) XRD patterns of the coatings.

The related EDX spectra (Figure 5a) clearly showed that all the elements from the target were transferred on the substrate. The intensity of the Si line was higher this time due to the fact that a large part of the signal came from the substrate, entirely made of silicon, which was also a proof of the films’ small thickness. The same bonds were identified in the FTIR spectra from Figure 5b as in the case of the target, with little changes in terms of a general trend and wavenumber values; indeed, the vibrations were not so well-defined this time, but it could be a consequence of the layer depth, in which the radiation was absorbed, or substrate presence, also contributing to the total intensity through its fingerprint. The coated samples were assessed from the crystallinity point of view by recording the XRD patterns at grazing incidence, ensuring a detectable signal coming from the film. According to the results shown in Figure 5c, both coatings had a quasi-crystalline level, explicable based on the existence of the diffraction halo at small angles, but also several diffraction maxima, not so sharp, suggesting small crystallites within a prevalent glassy matrix. The attempts to assign the major peaks to certain crystalline phases were doomed to failure, probably because of the system complexity. Our assumptions went to several types of SiO_2_, knowing the wide variety of distinct polymorphs that it has, or a complex/non-stoichiometric silicate. Analyzing the achieved intensities, a slightly more crystalline material was obtained at 300 °C, which was to be expected. The presence of a thin layer of SiO_2_ at the surface of the silicon substrate, as a known fact for the commercial product, had nothing to do with the aspect of the XRD patterns since similar films were deposited on titanium plates, and the obtained result in terms of crystallinity was identical.

Overall, we considered that the most determinant parameter was laser wavelength (355 nm), which provided insufficient energy for complete crystallization, but enough for promoting a short-range ordering. Since the few maxima were shaping up from the base halo, they could be a fingerprint of (SiO_4_)^4−^ tetrahedra connection following a certain geometrical rule. The best match was found for silicon dioxide (SiO_2_) with cubic symmetry (ICDD 00-084-0384), bringing up that all diffraction peaks were shifted to the right compared to the standard lines; this could be a consequence of crystalline network doping with other cations provided by the system itself.

### 3.3. Coatings’ Biological Evaluation

**Figure 6:**
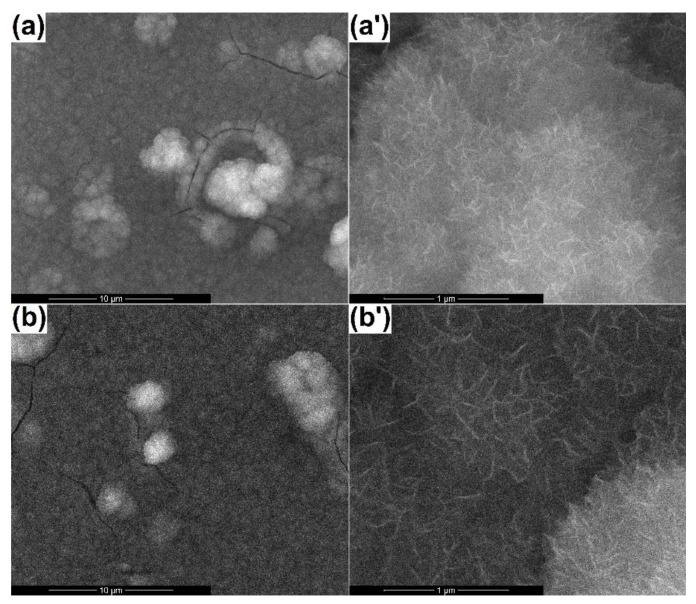
SEM images of the coatings processed at (**a**-10,000× and **a’**-100,000×) room temperature and (**b**-10,000× and **b’**-100,000×) 300 °C, after immersion in simulated body fluid (SBF) for 28 days.

One of the tests performed to determine the biological response of the coated samples was to study their behavior upon immersion in simulated body fluid (SBF) for 28 days; the testing solution was prepared according to Kokubo [33]. The resulting surfaces were subjected again to SEM investigation to identify potential changes in their morphology as an outcome of apatite deposition. The corresponding images are presented in Figure 6. In both cases, the presence of fluffy aggregates made of radially packed nanosheets was obvious on the whole analyzed area, but their quantity was rather different, more precisely was higher when operating at room temperature, as revealed by the low magnification images. Moreover, at higher magnification, the spherical entities occurring on the films’ surface appear to be slightly less dense, airier, and softer due to lower material content. Extended cracks were also visible, possibly because of the tensioning that emerged during their growth or contraction during the drying process; these could represent, as well, proofs of considerable thickness, meaning a fast and pronounced capacity of mineralization. Similar shells were reported at the surface of 45S5 bioglass-derived glass-ceramics after soaking in SBF for different periods of time; even though the entities are made of fine grains instead of thin sheets, their spherical shape and way of settlement is identical [37,39].

**Figure 7:**
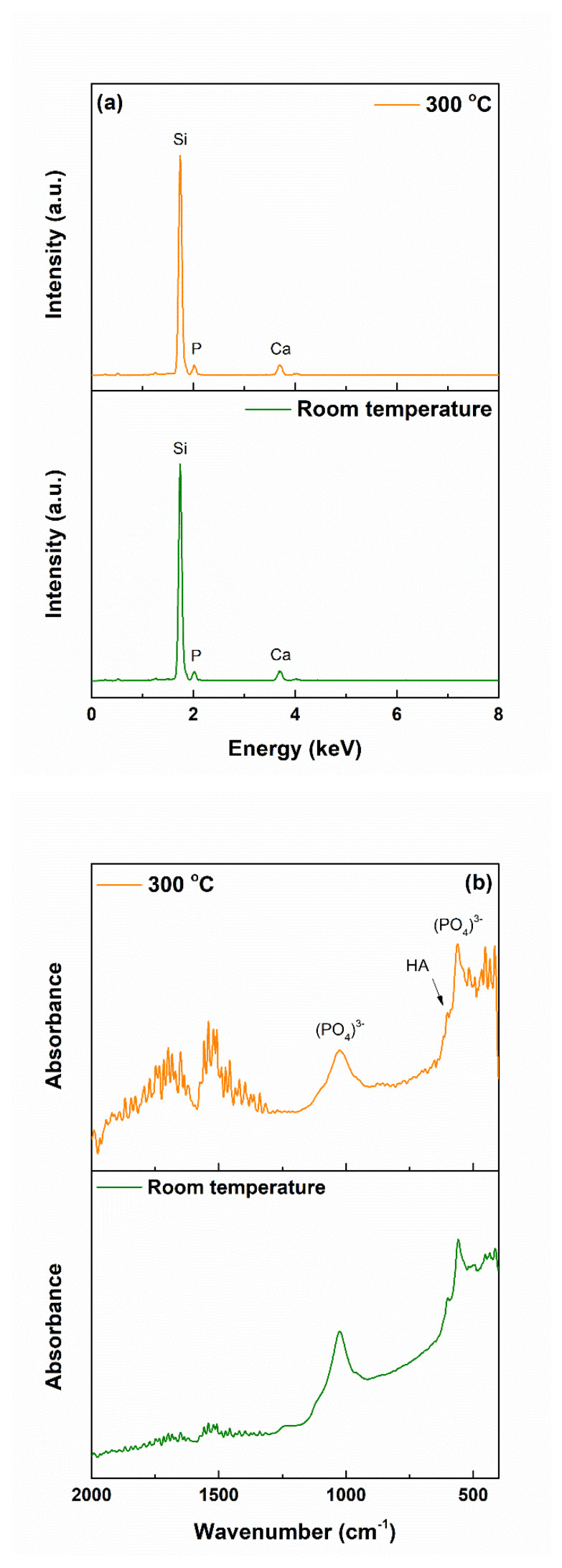
(**a**) EDX spectra and (**b**) FTIR spectra of the coatings, after immersion in SBF for 28 days.

The above statements were sustained by the EDX and FTIR spectra (Figure 7). In the first set of curves, the signals associated with Ca and P became dominant and shielded the contributions of the elements found in the films, highlighting the quantitative development of the apatite layer. The second set of spectra brought similar information, namely, the profiling of new vibrational characteristics, which hid the former succession of bands, characteristics that were linked with (PO_4_)^3−^ group, typical of calcium phosphates; more than that, a shoulder could be noticed at approximately 600 cm^−1^, a well-known fingerprint of hydroxyapatite (HA) [35,41].

As the essential requirement for an artificial material to bond to the living hard tissue, the formation of bone-like apatite on its surface can be reproduced in vitro by immersion in an acellular fluid with ion concentrations nearly equal to those in human blood plasma [42]. The mechanism behind this process resides in the dissolution of calcium cations from the corresponding glasses, where they are not so well-rooted as in a crystalline compound, the fact that generates an increase in supersaturation of the environment with respect to apatite; in the next stage, the hydrated silica occurring on the surface provides specific favorable sites for apatite nucleation, the easily formed nuclei growing spontaneously on account of the consumption of calcium and phosphate ions from the surrounding fluid [43].

**Figure 8:**
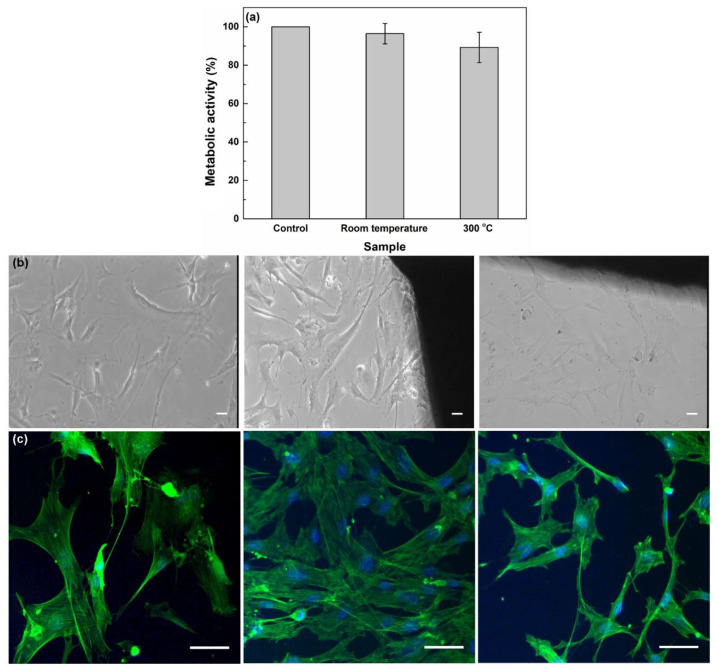
(**a**) Cell metabolic activity, (**b**) optical microscopy images (10× objective), and (**c**) fluorescence microscopy images (40× objective) of BJ cells grown on the coatings (middle panel-room temperature, right panel-300 °C) as compared to the control cells grown on a glass slide (left panel). The scale bar is 10 μm for all images. Statistical analysis was performed using one-way analysis of variance with Dunnett’s multiple comparison test. No statistical difference was found for the treated samples as compared to the control.

The next step was to estimate the response of BJ cells placed in contact with the two developed coatings. The cell metabolic activity test showed that the cells cultured on the investigated surfaces were not influenced, indicating that the layers were compatible and did not affect their development (Figure 8a). A slight superiority appeared in the case of the film deposited at room temperature. The images generated through transmission (Figure 8b) and fluorescence (Figure 8c) microscopy were in concordance with the MTT assay. Growing the cells in the presence of the samples did not change the morphology of the cells and did not decrease the number of cells found on the surfaces or around them. Both the actin filaments and nucleus were intact, but on comparing the displayed images, an appreciable increase in the number of cells was noticed for the same coating, grown in environmental conditions; the cells presented extensions and a higher degree of proliferation, meaning that a lower deposition temperature favored both higher bioactivity through the less rigid chemical structure and higher biocompatibility via a suitable roughness. Our in vitro studies were in concordance with previous reports on bioactive glass-ceramic with similar composition, which also showed good biocompatibility to human cells [44,45,46,47].

To have a better image of the biological impact of the obtained coated specimens, further studies are needed, both on BJ cells, as well as on bone cells. The materials need to be evaluated, according to the recommended methods (ISO 10,993, etc.), studying in vitro, more in-depth, the metabolic impairment by monitoring the enzymatic activity and/or metabolite concentration. Following the in vitro studies, the tests can be extended to in vivo studies, through which one can investigate the systemic and cytotoxic effects of the developed layered structures.

Considering the described study, the following research limitations can be formulated: the approached oxide system is a complex one, and numerous compositional points should be tested in order to identify the best one for medical purposes; PLD is a technique that theoretically addresses flat and stretched surfaces, raising problems in the case of implants with complex geometric shapes; more advanced biological tests performed on different cell cultures are needed to validate the suitability of the proposed materials for clinical applications.

## 4. Conclusions

Mineral coatings belonging to SiO_2_‒P_2_O_5_‒CaO‒MgO‒Na_2_O oxide system, a variation of the well-known 45S5 commercial glass composition, were obtained by fabricating a target and depositing its material in the form of thin layers by pulsed laser deposition. The evolution from gel to powder and then to target and films was followed by means of compositional, structural, and morphological characterization. The substrate temperature during film growth was varied in order to investigate its influence on biological properties. Thus, the sample achieved at room temperature showed a better capability of mineralization and turned out to be better accepted as support by the human fibroblast BJ cells.

Such biocompatible and bioactive mineral composition can be employed in the form of two-dimensional structures for encapsulating the inert metallic or ceramic supports in a body fluid responsive shell that could ensure rapid and efficient osseointegration of the load-bearing artificial implants or as fillers/scaffolds designed for superior bone regeneration.
